# Brain Mitochondria, Aging, and Parkinson’s Disease

**DOI:** 10.3390/genes9050250

**Published:** 2018-05-11

**Authors:** Mario Rango, Nereo Bresolin

**Affiliations:** 1 Parkinson’s Disease Center, Neurology Unit, Department of Neuroscience and Mental Health, Fondazione IRCCS Ca’ Granda Ospedale Maggiore Policlinico, 20100 Milan, Italy; nereo.bresolin@unimi.it; 2 Università degli Studi of Milan, 20100 Milan, Italy

**Keywords:** mitochondria, aging, Parkinson’s disease, genes, oxidative phosphorylation

## Abstract

This paper reconsiders the role of mitochondria in aging and in Parkinson’s Disease (PD). The most important risk factor for PD is aging. Alterations in mitochondrial activity are typical of aging. Mitochondrial aging is characterized by decreased oxidative phosphorylation, proteasome activity decrease, altered autophagy, and mitochondrial dysfunction. Beyond declined oxidative phosphorylation, mitochondrial dysfunction consists of a decline of beta-oxidation as well as of the Krebs cycle. Not inherited mitochondrial DNA (mtDNA) mutations are acquired over time and parallel the decrease in oxidative phosphorylation. Many of these mitochondrial alterations are also found in the PD brain specifically in the substantia nigra (SN). mtDNA deletions and development of respiratory chain deficiency in SN neurons of aged individuals as well as of individuals with PD converge towards a shared pathway, which leads to neuronal dysfunction and death. Finally, several nuclear genes that are mutated in hereditary PD are usually implicated in mitochondrial functioning to a various extent and their mutation may cause mitochondrial impairment. In conclusion, a tight link exists between mitochondria, aging, and PD.

## 1. Introduction

High energy requirements tissues such as the brain are highly dependent on mitochondria. Mitochondria are intracellular organelles deriving and storing energy through the respiratory chain by oxidative phosphorylation [1,2]. In a single neuron, hundreds to thousands of mitochondria are contained. Mitochondria have several copies of the mitochondrial genome that consists of a 16.5 kb circular DNA molecule [2,3] that, in turn, provides the template for 13 essential proteins of the respiratory chain. In a mitochondrion, there are more than 900 proteins and most of them are encoded by the nuclear genome [2,3]. Several diseases are caused by inherited mutations in mitochondrial DNA (mtDNA) [4]. If mutations occur in one single cell, all copies of the mitochondrial genome are called homoplasmic while these copies are otherwise called heteroplasmic. Not inherited mtDNA mutations are called somatic mutations and appear over time. Heteroplasmic mtDNA mutations derive clonally from expansion of single mitochondria with a resulting normal and respiratory-deficient mitochondria mixture within the same cell. Mutated mtDNA replication is better when compared to wild-type mtDNA, which facilitates its clonal expansion. Once mutated mtDNA reaches at least 60%, the cell will have deficient respiration and will accumulate additional mtDNA mutations until cell death [3,4].

Somatic mtDNA mutations are important in aging and disease such as Parkinson’s disease (PD) [5]. PD is a neurodegenerative common movement disorder with bradykinesia, rigidity, and resting tremor [5]. PD results mostly from the loss of dopaminergic neurons in the substantia nigra (SN) pars compacta (pc) region [5]. SN dopaminergic neurons are lost in an age and mitochondrial dysfunction related way [6,7].

When compared to other neurons, SN dopaminergic neurons have more mtDNA deletions, which is shown by two independent studies [6,7]. In these two studies, both in SN neurons of aged subjects and of subjects with PD, the load of mtDNA mutations paralleled the deficiency of the respiratory chain, which suggests that a common cell mechanism is at play in the two conditions.

## 2. Mitochondrial Activity in Aging

Aging, at the cell level, is an increasingly incapacity to recycle organelles and macromolecules [8,9].

In aging, proteasome activity decreases, autophagy is impaired, and mitochondrial dysfunction emerges with an activity decrease of oxidative phosphorylation, beta-oxidation, and Krebs cycle [10] as well as an increase of oxidative stress that, in turn, damages mitochondria [10].

Mitochondria DNA is very vulnerable [10]. The aging process is tightly linked to mtDNA deletions and point mutations and to reactive oxygen species (ROS). Additionally, mtDNA deletions and point mutations accumulate over time. This leads to energetics impairment, increased ROS production, mtDNA lesions, and the decline of mitochondrial respiration.

ROS target mtDNA since there are no histones and efficacious proofreading systems, this brings more point mutations and deletions [10]. In the mitochondria, deletions expand clonally over time and their number correlates linearly with the cell age.

Mitochondria produce ROS through their several electron carriers but, at the same time, have anti-oxidative capacities. The overexpression of mitochondrial antioxidant enzymes prolongs life [10] both in *Drosophila* and in mice.

It has been shown that, in old subjects, human frontal cortex oxidative stress damages genes involved in mitochondrial function [10].

In a primate model of aging [11], basal ganglia senescence was under lied by altered mitochondrial function and motor decline. In this model, older animals had minor motor activity than younger animals. In aged monkeys, ATP synthesis was reduced in SN and putamen (PUT). Additionally, pyruvate dehydrogenase activity and calcium buffering capacity were decreased in PUT. A decline in mitochondrial and motor function in the basal ganglia were correlated [11].

## 3. Aging, Mitochondria, and Parkinson Disease

Aging is the most important risk factor for PD [8,12,13]. In PD as in aging [14], there are similar changes in mitochondria such as reduced activity of complex I and IV, increased mtDNA deletions, swollen neuronal mitochondria, and reduced activity of PGC-1_ in the SNpc [15]. PGC-1_ is a transcriptional co-activator that regulates the expression of nuclear genes involved in mitochondrial genesis and oxidative phosphorylation and controls genes involved in the cell wear to oxidative stress.

### 3.1. Mitochondrial Dysfunction within Substantia Nigra Neurons

In about half of individuals, those who are older than 85 have mild parkinsonian signs [16]. Old subjects have neuronal loss. Specifically, within the SN, the dopaminergic neurons of the pars compacta are lost and this brain region also shows more pathological changes with normal aging than any other region. The pathological distinctive feature of PD is the loss of dopaminergic neurons in the SNpc [16]. 1-Methyl-4-phenylpyridinium (MPP+) causes parkinsonism and SN cell loss by inhibiting complex I [17,18]. Complex I activity and protein expression are decreased in brains of PD subjects [19,20]. SN neurons are very sensible for mitochondrial dysfunction, which causes loss of SN neurons.

All in all, reviewed data suggests that PD may be a local expression of aging on cell populations which, by their characteristics (high number of mitochondria as well as synaptic terminals and unmyelinated axons) are highly vulnerable to the agents promoting aging.

### 3.2. Respiratory Deficiency

The inhibition of complex I cause parkinsonism and its inhibitors, MPP+ and rotenone, cause death of SN neurons [17,21]. Respiratory deficiency is characterized by a decline in the complex IV, which is shown by cytochrome c oxidase/succinate dehydrogenase (COX/SDH) coloring [22]. In PD patients, around 3% of SN neurons are cytochrome c oxidase (COX) deficient with a few studies reporting up to 30% COX deficiency in some cases [6,7] when compared to 1% in age-matched subjects. Respiratory impairment brings impaired ATP synthesis. Compromised production of ATP has been shown in PD brain tissues with minor pathological involvement and with high mitochondrial activity such as the visual cortex [23].

### 3.3. mtDNA Mutation Load

In PD SN, when mtDNA deletions reach 50%, a respiratory (COX) deficiency is detected [6,22]. The mtDNA deletion percentage reaches its maximum in SN followed by [24,25,26,27,28] the basal ganglia, cerebellum, and cortical areas. mtDNA deletions appear during mtDNA damage repair [29] as a consequence of the oxidative environment. In mice with deficient mtDNA polymerase (*POLG* gene), alterations of mtDNA integrity brings decreased dopaminergic neurons and an aging phenotype [30,31] together with osteoporosis, kyphosis, weight loss, and increased somatic mtDNA mutations [31]. Additionally, the knock-out of the mitochondrial transcription factor A (TFAM) within dopaminergic neurons causes progressive parkinsonism, the accumulation of protein inclusions, and dopaminergic neuron loss together with decreased mtDNA expression [30]. In old Twinkle mutant mice [32], it has been shown that motor defects are present together with reduced SN neurons and accumulation of mtDNA.

### 3.4. Genes and Key Mitochondrial Processes

Alterations in genes implied in early onset PD can cause mitochondrial changes. For example, in *Drosophila* and cell cultures, the knockout of *PINK1*, Parkin, Alpha-synuclein (Alpha-syn), and *DJ-1* alters mitochondrial morphology [33,34,35,36,37] in several ways. Alpha-syn mutations change mitochondrial distribution and ultrastructure [36]. *PINK1* and *Parkin* mutations cause modification of the mitochondrial electron density and fragmentation of the mitochondrial network [33,34]. *DJ-1* loss is accompanied by mitochondrial fragmentation plus decreased mitochondrial membrane potential [38,39].

Leucine-rich repeat kinase *(LRRK2*) mutation also causes mitochondrial changes [40].

Calcium is important to keep pace making activity of the SN neurons and its handling depends on several genes linked to mitochondria.

Mitofusin 2 (*mfn2*) deals with handling of mitochondrial calcium and mitochondrial fusion and is crucial for the integrity of SN-to-striatum projections [41]. *DJ-1* modulates mitochondrial calcium handling as well as morphology [42]. Overexpression of *Parkin* potentiates the mitochondrial calcium handling [43].

Mitochondrial degradation through mitophagy is associated with mitochondrial membrane potential (ΔΨm) loss [44]. Mitophagy depends on the dissipation of ΔΨm [44] and ΔΨm orientates directionality of neuronal mitochondria. Most of the mitochondria with a low membrane potential are transported towards the cell body [45]. *PINK1* and *Parkin* are implicated in mitophagy through the targeting and degradation of mitochondria. In addition, the interaction of *PINK1* and *Parkin* seems to facilitate the turnover of respiratory chain components [46].

### 3.5. Mitochondria and Alpha-Synuclein

Lewy body characterizes PD [5] and contains Alpha-syn. Misfolding and accumulation of the Alpha-syn protein are hypothesized to be two main mechanisms in the pathogenesis of PD.

There is still little understanding of the interaction between Alpha-syn and mitochondria. Alpha-syn enters the mitochondria in an energy-dependent way [47]. Cardiolipin facilitates the interaction of Alpha-syn with the mitochondrial membranes. Alpha-syn has antioxidant property through cyclic oxidation/reduction of methionine residues in the N-terminal. Oxidation is located in the membrane with peroxidized lipids close to the protein while methionine is reduced in the cytosol.

The strongest toxic effect of Alpha-syn is right in the mitochondria. Its accumulation within mitochondria causes complex I malfunctioning, increased ROS synthesis, and reduced ΔΨm [48,49,50] that, in turn, are likely to exacerbate the mitochondrial impairment present in old SN neurons.

Alpha-syn also impairs the mitochondria delivery and transport and is characterized by modification of the mitochondrial cristae in cell culture. In addition, rotenone-dependent toxicity is increased by overexpression of mutated Alpha-syn while, conversely, Alpha-syn knockout gives resistance to 1-methyl-4-phenyl-1,2,3,6-tetrahydropyridine (MPTP) intoxication in mice.

Lastly, accumulated Alfa-syn interacts with motor proteins, which reduces axonal transport and alters the distribution of mitochondria along the neuron [51].

## 4. Mitochondria and the Ubiquitin Proteasome System in Aging and Parkinson Disease

Malfunctioning of the mitochondria and of the ubiquitin proteasome system (UPS) characterizes the aging process as well as several age-related diseases such as PD [52].

The UPS keeps mitochondrial homeostasis by controlling the proteome and mitophagy. Mitochondrial dysfunction alters homeostasis of cellular proteins through oxidative damage. In models, proteasome activation improves the aging process by increasing lifespan. In PD, it is difficult to isolate UPS impairment from mitochondrial dysfunction.

## 5. Mitochondrial Diseases and Parkinson Disease Symptoms

SN changes are more tightly associated with acquired rather than inherited mitochondrial defects [50]. In patients with mitochondrial disorders, extrapyramidal features are rare. However, there are reports on the association between Parkinsonism and *POLG* mutations [53,54,55,56]. *POLG* is the most common inherited mutation in patients with parkinsonism and mitochondrial diseases. However, sometimes parkinsonism is associated with point mutations of other genes [57,58].

The haplogroups K, J, and possibly T and superhaplogroup JT protect against PD. However, this is only within European populations [59,60,61]. The superhaplogroup HV has been associated with a risk of developing PD with advancing age [59].

## 6. Latest Findings

New evidence on mitochondria, age, and PD has emerged in the last five years.

Popa-Wagner et al. [62] have shown that the turnover rates for DMN1l and FIS1, which are proteins implicated in mitochondrial biogenesis, mitophagy, and fission go in opposite directions in the cerebellum of 22-month-old C57BL6j mice when compared to three-month-old mice. Unlike previous studies that have reported decreased turnover rates for the mitochondrial respiratory complexes of aged rodents, the authors found increased turnover rates for mitochondrial proteins of the oxidative phosphorylation chain in the aged mice when compared to young mice.

Cooper et al. [63] investigating the relationship between mitochondrial function, dopamine neuronal dysfunction, and death for *pdr-1*/*PRKN*, *pink-1*/*PINK1*, and *djr-1.1*/*DJ-1* found that *pdr-1* and *pink-1* mutants had deficitary dopamine-dependent behaviors while *djr-1.1* mutants showed an increased sensitivity to oxidative stress. Additionally, they found that *djr-1.1* mutants exhibit increased mitochondrial fragmentation together with a decreased rate of oxidative phosphorylation and ATP levels and that *pdr-1* and *pink-1* mutants showed an accumulation of dysfunctional mitochondria with age, which leads to activation of the mitochondrial unfolded protein response (mitoUPR). By preventing the upregulation of the mitoUPR, they obtained decreased lifespan and dopamine neuronal loss.

Hauser et al. found [64] that *DJ-1* knockout in both rat and mouse brains results in an age-dependent accumulation of hexokinase 1 in the cytosol (away from its usual location at the mitochondria) with subsequent activation of the polyol pathway of glucose metabolism in vivo. In their work, dissociation of hexokinases from mitochondria inhibited the *PINK1*/*parkin* pathway, which suggests that hexokinases are an important link between three major genetic causes of early onset PD.

Geldenhuys et al. [65] showed that loss of mitoNEET (CISD1), which is an iron-sulfur containing protein, regulates mitochondrial bioenergetics and results in mitochondrial dysfunction and loss of striatal dopamine and tyrosine hydroxylase. Mitochondria from mice lacking mitoNEET were dysfunctional with elevated ROS and reduced ATP synthesis. In knockout mice, gait analysis revealed a shortened stride length and decreased rotarod performance consistent with the loss of striatal dopamine. This suggests that mitoNEET KO mice exhibit many of the characteristics of early neurodegeneration in PD.

Song et al. [66] have shown that selective overexpression of the stress protein in heme oxygenase-1 (HO-1) in astrocytes of GFAP.HMOX1 transgenic mice between 8.5 and 19 months of age results in nigrostriatal hypodopaminergia and mitochondrial damage/mitophagy associated with locomotor incoordination and stereotypy. These GFAP.HMOX1^8.5–19m^ mice are affected by parkinsonism unlike younger GFAP.HMOX1^0–12m^ mice who have schizophrenia-like features. This depends on whether the glial HO-1 response is engaged prior to or following the maturation of dopaminergic circuitry.

Genetic deletion of Sirtuin-3 (*Sirt3*), which is a mitochondrial deacetylase, increases oxidative stress and decreases the membrane potential of mitochondria in SNpc dopaminergic neurons. Studies from Shi et al. [67] have suggested that an age-related decline in Sirt3 protective function is a major factor underlying increasing mitochondrial oxidative stress and loss of SNpc dopaminergic neurons in PD.

Stevens et al. [68]: 11696-701 have shown that, in a mouse model, adult knockout of parkin leads to an age-dependent loss of dopamine neurons and decreases in brain mitochondrial size, number, and protein markers in line with a defect in mitochondrial biogenesis.

In a mouse study, Jiang et al. [69] have shown that mitochondrial functions, mitochondrial fusion/fission-related proteins, and autophagic proteins are more severely affected by MPTP treatment in the elderly than in the young.

In humans, Cabre et al. [70] have shown that protein oxidative and glycoxidative damage significantly increases during human brain aging with a breakpoint at 60 years old together with a decrease in the content of the mitochondrial respiratory chain complex I-IV.

## 7. Conclusions

There is a link between mitochondria, aging, and PD. Several mitochondrial changes are shared and overlap in aging and PD. However, more research still has to be completed to fully elucidate the exact mechanisms underlying and linking the two conditions.
